# Dermoscopy of actinomycetoma

**DOI:** 10.1016/j.jdcr.2026.09.006

**Published:** 2026-09-10

**Authors:** Kenza Khachani, Meryeme Marhraoui, Kaoutar Znati, Mariame Meziane, Nadia Ismaili, Laila Benzekri

**Affiliations:** a Department of Dermatology, Mohammed V University, Ibn Sina University Hospital, Rabat, Morocco; b Department of Pathology, Mohammed V University, Ibn Sina University Hospital, Rabat, Morocco

**Keywords:** Actinomyces, actinomycetoma, dermoscopy

## Clinical presentation

A 43-year-old man, living in a rural environment, with no significant past medical history or trauma, presented with painful papules and nodules of the right foot 3 years ago. These lesions had rapidly increased in size over the past year, despite previous surgical excision and empirical oral antibiotic therapy elsewhere. Examination revealed multiple erythematous and violaceous papules and nodules on the medial foot arch, without discharge or granules (Fig 1). Foot X-ray was normal, while MRI revealed multiple soft tissue micro-abscesses extending to muscles, without bone involvement (Fig 2).

**Fig 1:**
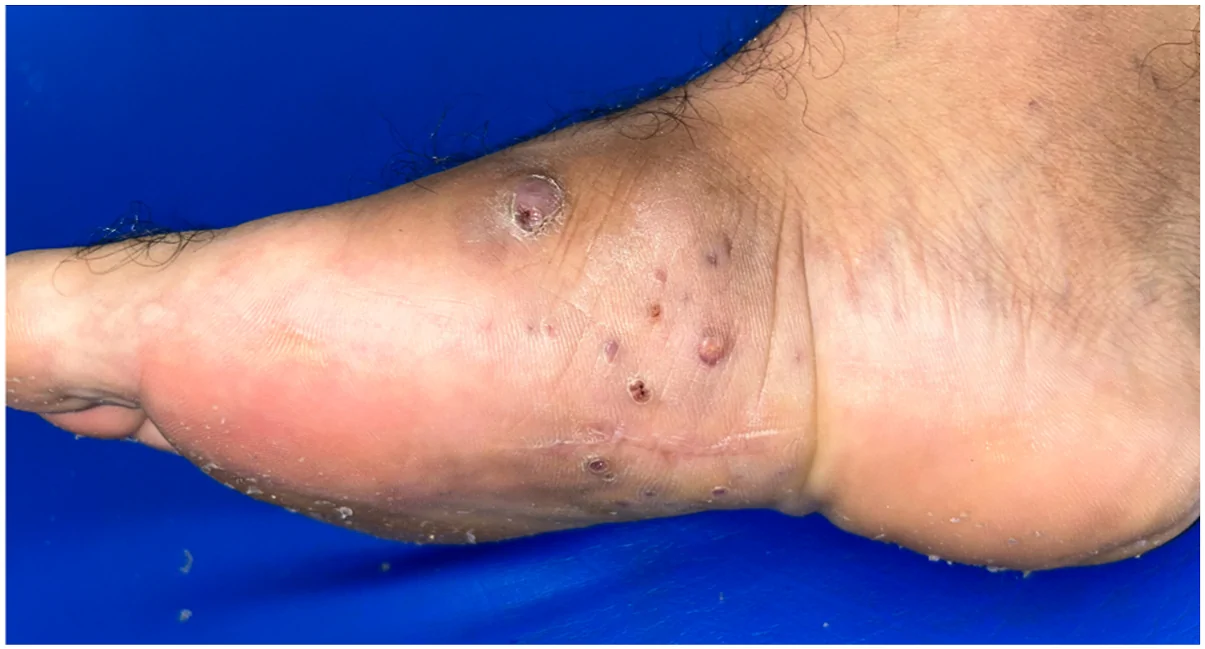
Erythematous papules and nodules of the medial foot arch.

**Fig 2:**
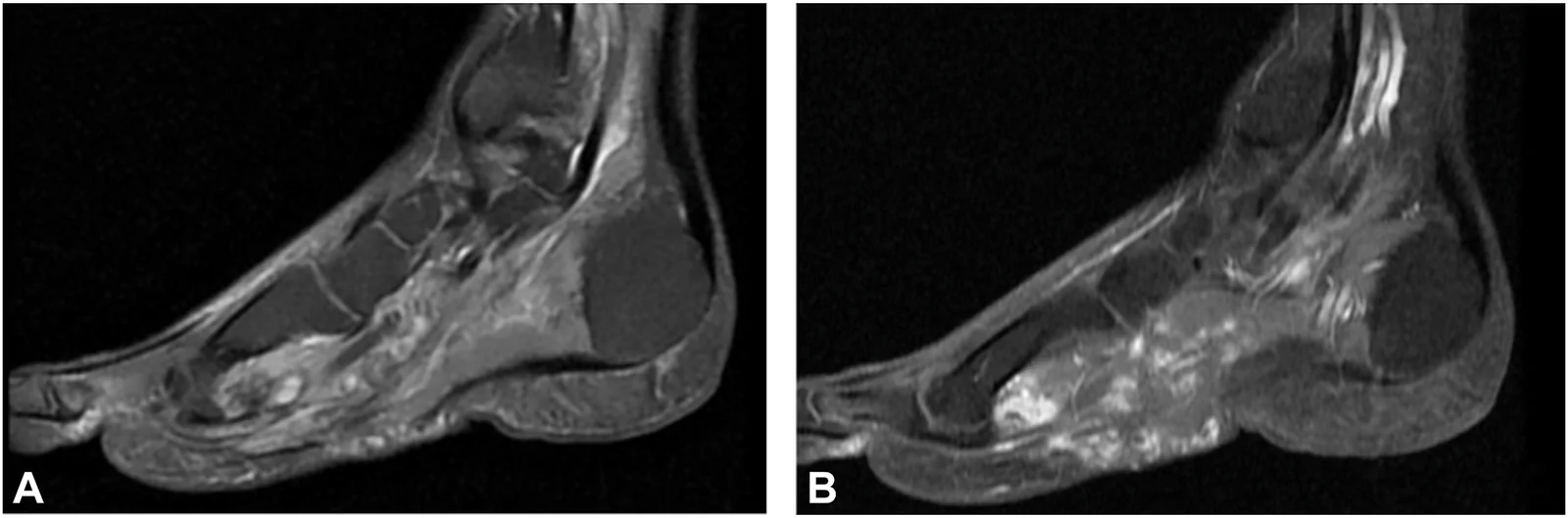
MRI of the right foot: sagittal slices revealing inflammatory infiltration with multiple collections that are hyperintense on proton density (PD) **(A)**; and enhanced by gadolinium **(B)**, extending to the plantar soft tissues, without bone or vascular lesions.

## Dermatoscopic appearance

Dermoscopy showed orange-yellow structures, brown-black globules and areas, white structureless areas, scales, and dotted vessels on an erythematous background. (Fig 3).

**Fig 3:**
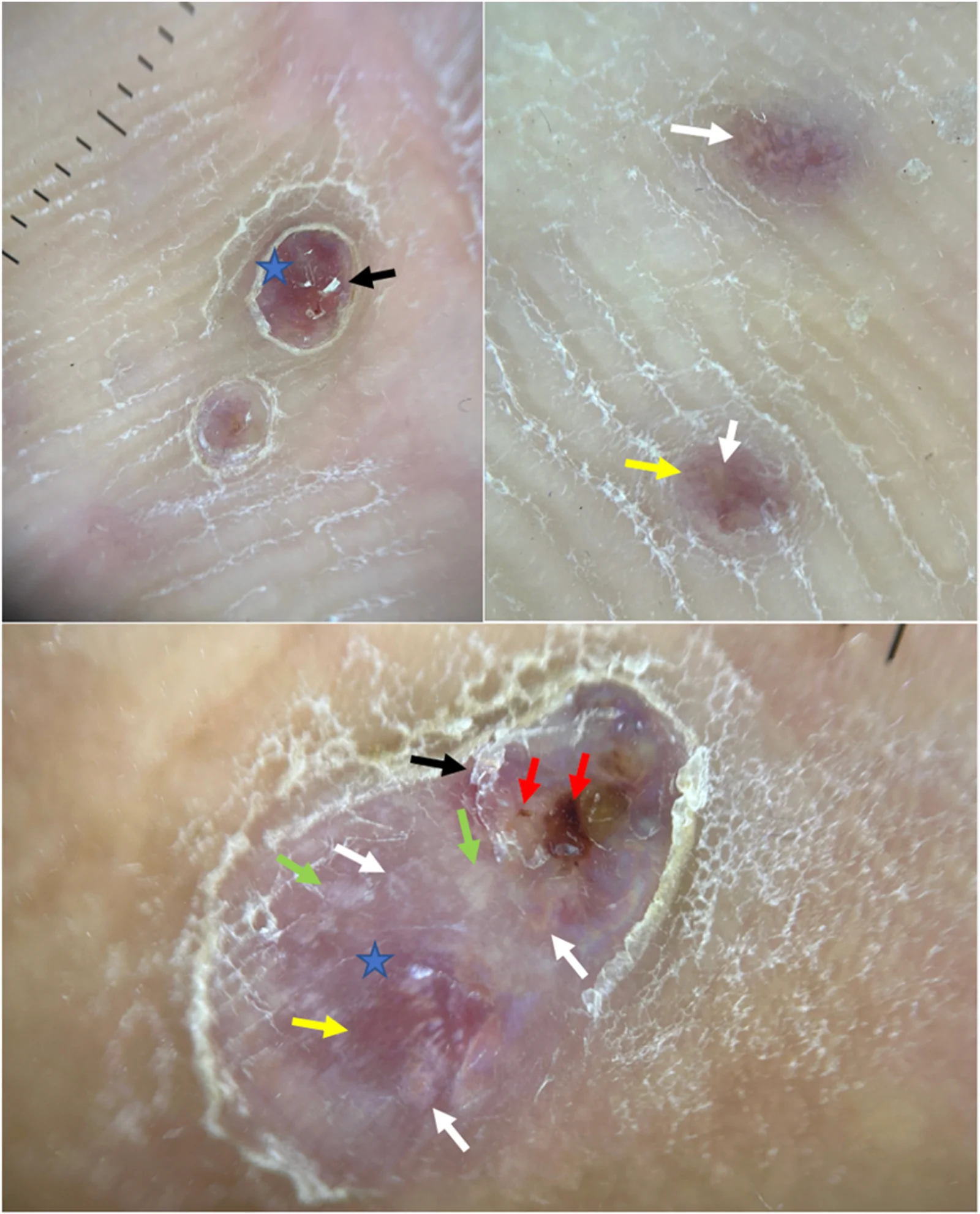
Dermoscopic features of actinomycetoma (DermLite DL5, polarized mode, ×10 magnification): Orange-yellow structures (*white arrow*), scales (*black arrow*), brown-black globules and areas (*red arrow*), white structureless areas (*green arrow*), dotted vessels (*yellow arrow*), Erythematous background (*blue star*).

## Histologic diagnosis

Histology revealed a polymorphic inflammatory infiltrate with neutrophilic nests surrounding basophilic granular structures composed of filamentous organisms consistent with actinomycetes (Fig 4), along with hyperplastic granulation tissue and capillaries. No tumoral lesion was identified. Immunohistochemistry staining was negative for HHV-8, ruling out Kaposi sarcoma.

Key message

Cutaneous actinomycetoma is a rare, chronic granulomatous infection caused by filamentous Gram-positive bacteria of the genus Actinomyces, which may progress to deeper structures if untreated. Dermoscopy, combined with clinical and histological features, helps achieve early diagnosis and prompt treatment, preventing deep progression. Dermoscopic features found in our patient were similar to those described in the literature: orange-yellow areas corresponding to granulomas, focal white scales corresponding to hyperkeratosis, and brown-black areas corresponding to dried blood.^1^ Direct examination, Gram staining, and bacteriological culture on the biopsy fragment were negative, and diagnosis relied on histopathology. Clinical improvement was noted with trimethoprim-sulfamethoxazole (40 mg/kg/d).

**Fig 4:**
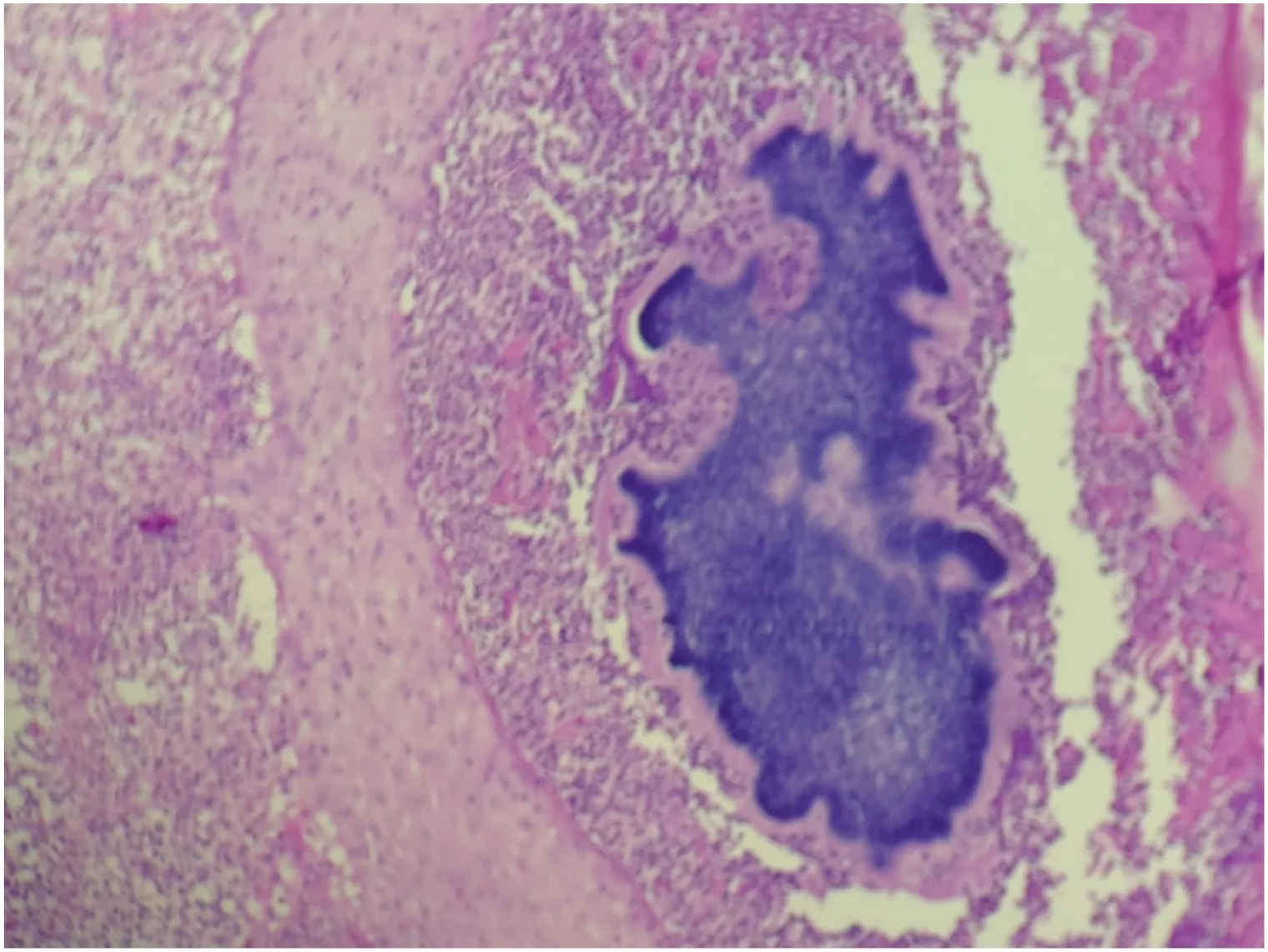
Histopathologic examination (hematoxylin-eosin stain, ×100) showing a basophilic actinomycotic granule surrounded by a dense polymorphous inflammatory infiltrate within the dermis, consistent with cutaneous actinomycetoma.

### Declaration of generative AI and AI-assisted technologies in the writing process

Not applicable.

## Conflicts of interest

None disclosed.

## References

[1] Ankad B.S., Beergoudar S.L., Nikam B.P. (2019). Dermatoscopy in actinomycetoma: an observation. Indian Dermatol Online J.

